# Blueprint of the distinct metabolite profiles of healthy pig heart chambers^☆^

**DOI:** 10.1016/j.jmccpl.2025.100462

**Published:** 2025-06-10

**Authors:** Retu Haikonen, Topi Meuronen, Ville Koistinen, Olli Kärkkäinen, Tomi Tuomainen, Gloria I Solano-Aguilar, Joseph F. Urban, Marko Lehtonen, Pasi Tavi, Kati Hanhineva

**Affiliations:** a Institute of Public Health and Clinical Nutrition, University of Eastern Finland, Kuopio, Finland; b Food Sciences Unit, Department of Life Technologies, University of Turku, Finland; c School of Pharmacy, University of Eastern Finland, Kuopio, Finland; d U.S Department of Agriculture, Agricultural Research Service, Northeast Area, Beltsville Human Nutrition Research Center, Diet Genomics and Immunology Laboratory, Beltsville, MD, United States; e A. I. Virtanen Institute for Molecular Sciences, University of Eastern Finland, Kuopio, Finland

**Keywords:** Metabolomics, Metabolomic profiles, Chambers, Healthy heart

## Abstract

The heart is one of the most studied organs, with physiological processes and disease research. While it is well-established that significant structural and functional differences exist between the chambers, most studies focus on only a single heart chamber, predominantly the left ventricle. This study aims to comprehensively characterise the chamber-specific metabolic profiles of all four heart chambers in a healthy animal model close to human metabolism, pigs. We employed liquid chromatography-mass spectrometry metabolomics to analyse the metabolite profiles of heart chambers in healthy pigs (*N* = 30) maintained on an ad libitum diet and housed under standard, non-stressed physiological conditions. Our findings reveal a higher energy demand in the left ventricle, as evidenced by elevated levels of electron transport chain-related metabolites such as NAD^+^ and FAD. Additionally, hexose-phosphates and several acylcarnitines exhibited chamber-dependent variations in abundance. The ventricles, particularly the left, demonstrated distinct redox states, with differential levels of glutathione and ascorbic acid, suggesting variations in oxidative stress across chambers. Furthermore, amino acids had chamber-specific abundance patterns, and ventricles showed an increased requirement for protein synthesis, likely associated with repair mechanisms following reactive oxygen species (ROS)-induced cellular damage. Our study reveals significant differences in the metabolic profiles across four heart chambers in healthy pig hearts, underscoring the metabolic heterogeneity of cardiac tissue. These findings highlight the necessity of investigating chamber-specific metabolic pathways to better understand heart functionality. Such insights could inform the development of more precise therapeutic strategies tailored to metabolic demands and functional roles in heart chambers.

## Highlights

• Significant metabolic differences measured in healthy pig heart chambers.
• Left ventricle displays elevated energy demand and oxidative stress markers.
• Amino acid patterns reflect chamber-specific metabolic and protein synthesis roles.
• Findings underscore metabolic heterogeneity even in the undamaged heart model.

## Graphical abstract

**Figure:**
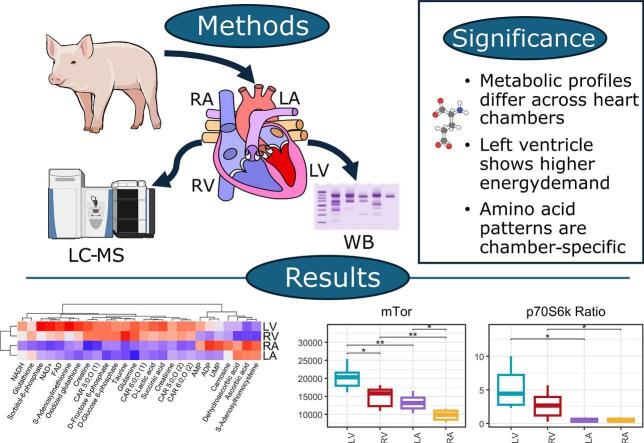

## 1 Introduction

The heart, a vital organ responsible for pumping blood throughout the body, relies on complex metabolic processes to sustain its continuous rhythmic contractions. It consists of distinct chambers, including the atria and ventricles, each exhibiting specialised metabolic characteristics reflecting their unique functional roles [1]. While the atria primarily manage blood inflow and optimise ventricular filling, the ventricles generate the force necessary to propel blood into systemic and pulmonary circulation. Different heart chambers are also subject to specific pathologies, many of which involve metabolic disturbances [2]. For instance, left ventricular hypertrophy is linked to conditions such as aortic stenosis [3,4], right ventricular dilation is often associated with pulmonary diseases [5,6], and atrial fibrillation is observed [7] meaning that the understanding of the metabolic differences between the heart chambers is crucial for advancing our understanding of the aetiology of heart diseases and subsequent diagnostics, treatment, and prevention.

The molecular mechanisms that regulate cardiac function are tightly controlled by an array of metabolites, which play essential roles in energy production and cellular signalling pathways [8]. Although the full spectrum of molecular mechanisms in cardiac tissue is not yet fully characterised, metabolomics offers a powerful approach to studying molecular differences across tissues, including investigation between heart chambers [9,10]. This approach enables a comprehensive analysis of metabolic processes, which is particularly valuable to better understand heart function [11,12]. Despite its potential, metabolomics has yet to be fully leveraged to explore chamber-specific metabolic differences in the heart, offering an opportunity to illuminate the intricate interplay of metabolic pathways underlying cardiac physiology.

The heart chambers differ significantly in workload, with the ventricles bearing a higher demand than the atria [13]. This functional disparity suggests distinct metabolic roles between chambers, leading us to hypothesise a higher abundance of energy-related metabolites in the ventricles. Metabolites like acylcarnitines, glucose, and amino acids are essential for myocardial energy metabolism, while amino acids and peptides are more supportive for heart muscle function [14,15]. Dysregulation in these pathways has been linked to cardiovascular diseases, including myocardial infarction and heart failure [16,17]. Our study aims to explore these chamber-specific metabolic profiles to provide insights that could eventually aid in developing more targeted treatments for heart disease.

This study investigates the metabolic signatures of the left and right atria and ventricles to characterise the molecular landscape of healthy pig heart chambers. The pig heart was selected as a model organism due to its close resemblance to the human heart in terms of metabolism, pressure dynamics, morphology, and, importantly, size, making it a more translational model than rodent hearts [18]. With this model, we explored the differences in metabolic profiles across heart chambers and further analysed gene expression of selected targets, protein levels, and pyruvate dehydrogenase activity. For the first time, these findings provide proof that chamber-specific metabolism manifests in healthy heart tissue of a large mammalian model, providing a foundational blueprint of heart chamber-specific metabolite profiles that can inform future mechanistic research and biomarker studies.

## 2 Methods

### 2.1 Animals, sample collection and preparation

Thirty pigs were kindly obtained from the experimental farm of the Beltsville Agricultural Research Center (BARC), Beltsville, MD at six weeks of age. Pigs were derived from boars and gilts from a four-way crossbred composite BX line (Duroc X maternal Landrace X terminal Landrace X Yorkshire) to be genetically similar to commercial swine. Pigs born at BARC are screened yearly for porcine reproductive and respiratory syndrome virus (PRRSV), influenza (H1N1 and H3N2), pseudorabies, and brucellosis by the Veterinary Services Group at the Beltsville Agricultural Research Center and have been negative for these infections. Weaned pigs were individually housed in stalls with a non-absorptive concrete floor surface and provided ad libitum access to water. Pigs were fed daily ad libitum with general pig chow enriched with different bread (Haikonen et al., manuscript). All protocols for animal use and euthanasia were reviewed and approved by the Beltsville Area Animal Care and Use Committee under Protocol no. #16-009 and were in accordance with the National Institutes of Health (NIH) guidelines for the Care and Use of Laboratory Animals following the standards established by the Animal Welfare Act.

All pigs were euthanised by intravenous injection with Euthasol (50 mg sodium pentobarbital/kg of body weight) (Virbac Animal Health, Inc., Fort Worth, Texas, USA) at the end of the study. Left ventricle, right ventricle, left atrium, and right atrium tissues from 30 pigs aged 16 weeks were immediately obtained after euthanasia. Tissue samples were snap-frozen in liquid nitrogen, stored at −80 °C and shipped on dry ice to the University of Eastern Finland for further analysis. All frozen samples were first pre-ground with liquid nitrogen cooled mortar and then tissues were weighed into precooled (−80 °C) homogeniser microtubes (OMNI International, Kennesaw, GA, USA, Cat.No 19-620) with 200 mg target weight. Tissues were treated with ice-cold 80 % methanol (CHROMASOLV™ LC-MS Ultra, Riedel-de Haën™, Honeywell, Seelze, Germany, Cat. No. 14262-2L) in a ratio of 600 μl solvent per 200 mg tissue. Samples were cryo-ground with precooled (+4 °C) Omni Bead Ruptor Elite homogenisers attached to the Bead Ruptor Cryo cooling unit for 30 s at 60 m/s. Then samples were incubated on a shaker (Heidolph Multi Reax) at 2000 rpm for 5 min at room temperature. All samples were centrifuged for 10 min at +4 °C (20.000 ×*g*), and the supernatant fractions were collected and diluted 1:3 of 80 % methanol before filtering with 0.2-μm centrifuge filter plate (Agilent, Santa Clara, CA, USA, Cat.No. A5969002) for 5 min at +4 °C (700 g). Samples were stored at −20 °C until analysis with liquid chromatography-mass spectrometry (LC-MS). The order of samples was randomised before analysis.

### 2.2 Instrumentation in metabolomics

The non-targeted metabolomic profiling was analysed using both reversed-phase (RP, Zorbax Eclipse XDB-C18, 2.1 μm 100 mm, 1.8 mm column, Agilent Technologies, Palo Alto, CA, USA) and hydrophilic interaction (HILIC, Acquity UPLC BEH Amide 1.7 μm, 2.1 × 100 mm column, Waters, Ireland) chromatography LC-MS metabolomics center (University of Eastern Finland, Kuopio). For RP mode, an ultra-high performance liquid chromatography (Vanquish Flex UHPLC system, Thermo Scientific, Bremen, Germany) coupled with high-resolution mass spectrometry (Q Exactive Focus, Thermo Scientific, Bremen, Germany) was used. For HILIC ultra-high-performance liquid chromatography (1290 Infinity Binary UPLC system, Agilent Technologies, Santa Clara, CA, USA) coupled to 6540 UHD Accurate-Mass Q-TOF MS (Agilent Technologies, Santa Clara, CA, USA). Both positive (ESI+) and negative (ESI-) data acquisition modes were used. The full description of the LC-MS instrument setup has been published previously; for HILIC [19] and RP [20]. Aliquots (20 μl) from all the samples were pooled to form a quality control sample (QC), which was injected at the beginning of the analysis and after every 12 samples.

With HILIC chromatography and Agilent QTOF instrument, the tandem mass spectrometry (MS/MS, data-dependent) analysis was used with three different collision-induced dissociation voltages (10, 20, and 40 V) and for RP and Thermo Orbitrap instrument as previously described [20]. Pooled QC samples and tissue-specific QC samples were used for MS/MS data acquisition.

Combined data from HILIC (positive and negative ionisation modes) and RP (positive and negative ionisation modes) analyses comprised 16,044 molecular features from four heart tissue types from the control diet and 4 different types of bread diet. There were a total of 4130 and 4105 molecular features obtained from HILIC, and 4939 and 2870 features from RP, in positive and negative ionisation modes, respectively.

### 2.3 Data extraction and identification

Raw data was processed through MS-DIAL software (version 3.96) for baseline filtering, baseline calibration, peak picking, identification, peak alignment, and peak height integration [21,22]. Peak picking parameters according to Klåvus et al. [19] were used, with the following changes, counts higher than 1000 and 250,000 counts were selected from centroid data and MS1 tolerances of 0.01 and 0.003 for QTOF and Orbitrap instruments data, respectively, and an alignment RT tolerance of 0.1 min was used.

Features were identified by utilising an in-house library maintained by the Metabolomics Center in Biocenter Kuopio. It has ca. 600 pure compound standards with retention time, the mass-to-charge ratio (*m/z*), and MS/MS spectral data. Additionally, publicly available mass spectrometry libraries were used. For example, METLIN (https://metlin.scripps.edu), MassBank of North America (MoNA, https://mona.fiehnlab.ucdavis.edu), Human Metabolome Database (HMDB, www.hmdb.ca), and LIPID MAPS (https://www.lipidmaps.org) metabolomics databases were used.

### 2.4 Pyruvate dehydrogenase

The activity of pyruvate dehydrogenase (PDH) was analysed accordingly with a specific Pyruvate Dehydrogenase Activity Assay Kit (Sigma-Aldrich MAK183). Briefly, tissue samples were homogenised in an ice-cold PDH assay buffer. PDH reaction mixes were prepared according to instructions. The standard solution was measured with 0 (blank), 2.5, 5, 7.5, 10, and 12.5 nmol/well, and the activity of duplicated samples was calculated based on the established standard curve.

### 2.5 Western blot analysis

Total proteins extracted from the chambers were used for Western blot analyses. For detection of p70 and mTor proteins together with their phosphorylated form, 50 μg of protein were run on 6 % and 8 % SDS-PAGE gels, respectively. Proteins were transferred to the nitrocellulose membrane and incubated overnight at 4 °C with primary antibodies against the mammalian target of rapamycin (mTOR) (Cell Signaling #2972), phospho-mTOR (Cell Signaling #5536), ribosomal protein S6 kinase beta-1 (p70 S6K) (Cell Signaling #9202), and phospho-p70 S6K (Cell Signaling #9205). HRP conjugated goat anti-rabbit IgG secondary antibody (Cell Signaling #7074) was incubated for 1 h at room temperature. Protein bands were visualised with Western Lightning Ultra (PerkinElmer Inc., Waltham, MA, USA) chemiluminescent substrate. Blots were imaged with the Chemidoc MP imaging system (Bio-Rad, Hercules, CA, USA). Quantification of proteins was done with ImageJ software, and Ponceau S staining (Sigma-Aldrich) was used as a loading control.

### 2.6 qPCR analysis

Total RNA was extracted from the heart chambers using TRIzol reagent (Invitrogen). RNA was reverse transcribed by using random hexamer primers and RevertAid Reverse Transcriptase (Thermo Scientific) and gene expression was analysed using TaqMan gene expression assays (Thermo Fisher Scientific). Quantitative measurements of mRNAs were performed with StepOnePlus Real-Time PCR System with SYBR Green Master Mix (Thermo Fisher Scientific) as recommended by the manufacturer. Sequences of PCR primers were designed based on the pig nucleotide sequences in the SScrufosa 10.1 genome. Each chamber group included six pigs in qPCR, PDH, and Western blot analyses. For qPCR, we used the following primers:

**Table:** 

Assay name	Sense primer	Anti-sense primer
susRPL3	CAAGGATGACTCTTCCAA	GTCTCCACAATAGTCACA
susRPS12	GGACGTTAATACTGCTCTA	CTCCACCAACTTGACATA
susSLC16A1	GAGGTCCTATCAGCAGTA	CGGTGTTACAGAAGGAAG
susSLC25A20	CCTCTCAAGAATCTGCTG	ACACCCTCTCTCATAAGA
susSLC2A4	GCTACCTAAGACAAGATG	CTCTGTTCAATCACCTTC
susB2M	CAAGGACTGGTCTTTCTA	GCAGCATCTTCATAATCTC

Housekeeping genes 18S and B2M were used to normalize expressions.

### 2.7 Statistical analysis in metabolomics

Before starting statistical analysis for metabolomics data, data were preprocessed with the notame package (0.0.7) similarly as previously demonstrated [19]. Briefly, missing values were assigned, features with lower than 70 % detection in QC samples were flagged, analytical drift was corrected, samples with low quality were determined with the quality assurance method [23] and flagged, and missing values were imputed by utilising a random forest approach [24]. Furthermore, features that had an abundance of <5000 in >70 % of samples were flagged.

All the data processing was carried out by the R package (4.0). No statistical methods were used to pre-determine sample sizes. Fold change was used to evaluate the difference in size between the chambers. Log(2) transformation was done accordingly. ANOVA and Welch's *t*-test were chosen to identify differentially abundant features between the tissues, and *t*-tests were calculated between LV-RV, LV-LA, RV-RA, and LA-RA. False discovery rate correction was done based on the Benjamini-Hochberg procedure.

## 3 Results

### 3.1 The metabolite profiles are most distinct between ventricles and atria

**Fig. 1:**
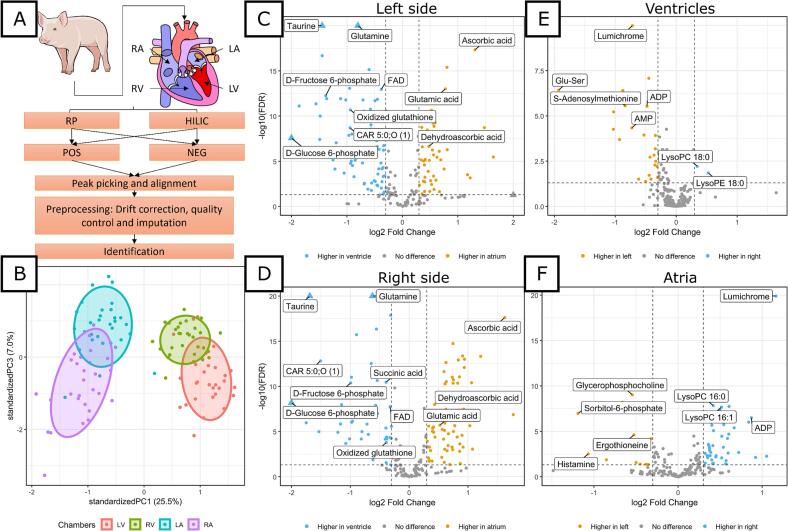
Differences between ventricles and atria on the left and right sides of the heart (*n* = 30 per group). (*A*) Overall description of the work. (*B*) Chambers differ in the PCA plot. The left ventricle (red) and right ventricle (green) cluster more distinctly from the left atrium (blue) and right atrium (purple). (*C–F*) Volcano plots show differences between conditions. Log2 fold change is plotted on the x-axis, and −log10(FDR *p*-value) on the y-axis. Horizontal lines indicate q < 0.05 (Welch's *t*-test, FDR-adjusted), and vertical lines indicate log2 fold change thresholds of ±0.3. (*C*) Left ventricle vs. left atrium. (*D*) Right ventricle vs. right atrium. (*E*) Left ventricle vs. right ventricle. (*F*) Left atrium vs right atrium. (For interpretation of the references to colour in this figure legend, the reader is referred to the web version of this article.)

Metabolite abundances were comprehensively measured across various compartments of the healthy pig heart. Importantly, the pigs maintained a stable body weight within the normal range throughout the study. Metabolomic profiling was performed using untargeted liquid chromatography-mass spectrometry (LC-MS), incorporating both hydrophilic interaction chromatography (HILIC) and reversed-phase (RP) chromatography, as illustrated in Fig. 1A. Metabolite identification was focused on molecular features with significant differences between chambers. Of the 203 identified metabolites, 171 exhibited significant differences (ANOVA FDR < 0.05) in abundance across the healthy pig heart chambers, together with numerous interesting unidentified compounds (Supplementary material online, Table S1). Furthermore, the PCA results demonstrate a clear separation between the chambers (Fig. 1B), highlighting distinct metabolite patterns, especially between ventricles and atria. The volcano plot further illustrates these differences, particularly in energy-related metabolites and amino acids, between the left ventricle and atrium (Fig. 1C), with comparable patterns observed on the right side of the heart (Fig. 1D). The most notable difference was the high levels of taurine and glutamine in the left ventricle. Additionally, there were low levels of ascorbic acid and glutamic acid in the left ventricle compared to the left atrium (Fig. 1C). Similarly, the right ventricle exhibited high levels of taurine and glutamine, whereas ascorbic acid was most abundant in the right atrium (Fig. 1D). While comparing the ventricles and atria, LysoPC 18:0 and LysoPE 18:0 were demonstrated to have higher abundance in the right ventricle (Fig. 1E). Moreover, LysoPE 18:0 and LysoPCs, such as LysoPC 16:0, 16:1, 18:0 and 18:3 were more abundant in the right atrium (Fig. 1F). Interestingly, lumichrome had a higher abundance in the left ventricle and right atrium (Fig. 1E, F). Still, similar types of metabolites, such as energy metabolism- and peptide metabolism-related, were observed to be differently abundant between left and right ventricles and atria.

### 3.2 Chamber-dependent energy metabolism differences

**Fig. 2:**
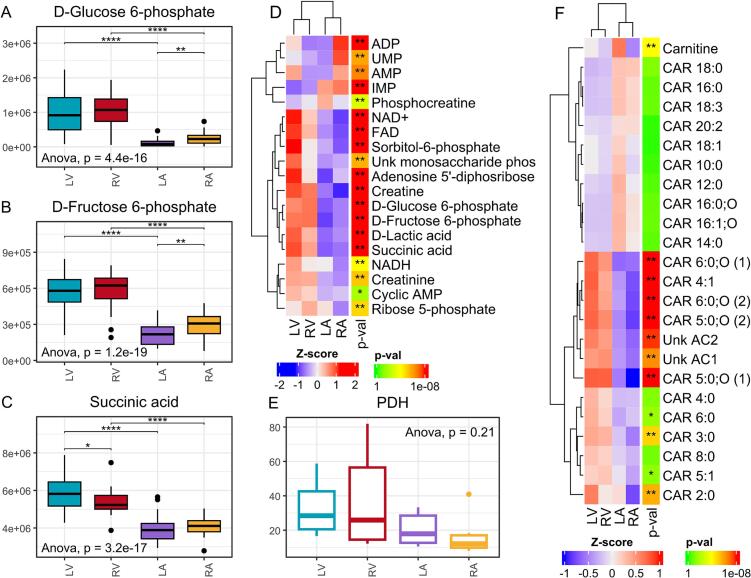
Energy production-related metabolites have different abundances in the chambers (n = 30 per group). (*A–C*) Metabolite abundances. (*, *p* < 0.05), (**, *p* < 0.01), (***, *p* < 0.001), and (****, *p* < 1e−04) in *t*-test, unadjusted. (*D*) Heatmap of energy metabolism-related metabolites. (*, p < 0.05), (**, q < 0.05) in ANOVA, unadjusted and adjusted, respectively. (*E*) Pyruvate dehydrogenase activity. (*F*) Carnitine and acylcarnitine abundance heatmap. (*, p < 0.05), (**, q < 0.05) in ANOVA, unadjusted and adjusted, respectively. Legend: LV = left ventricle, RV = right ventricle, LA = left atrium, RA = right atrium, ADP = adenosine diphosphate, UMP = uridine monophosphate, AMP = adenosine monophosphate, IMP = inosine monophosphate, NAD+ = Nicotinamide adenine dinucleotide, FAD = flavin adenine dinucleotide, NADH = NAD+ + hydrogen, PDH = pyruvate dehydrogenase, CAR = acylcarnitine, and Unk = Unknown.

Our analysis demonstrated a consistently higher relative abundance of hexose-phosphates in ventricles compared to the atria (Fig. 1C, D). Glucose-6-phosphate showed a fold change of 8.31 on the left side (ventricle vs. atrium) and 4.29 on the right side, while fructose-6-phosphate had a fold change of 2.61 on the left side and 1.99 on the right side, respectively (Fig. 2A, B). Furthermore, other metabolites directly related to energy production, such as succinic acid, creatine, and NAD^+^ have a higher abundance in ventricles compared to atria (Fig. 2C, D). Lastly, AMP and ADP abundances are significantly higher in the left ventricle compared to the left atrium (Fig. 2D).

The differences between ventricles and atria suggest that ventricles possess greater metabolic reserves for energy production, ensuring an adequate supply to meet their higher energy consumption. Additionally, the higher abundance of hexose-phosphates in the ventricles likely reflects their greater energy demand compared to the atria. To further characterise the differences in glycolysis-related energy production pathways between heart chambers, we assessed the activity of PDH, a key enzyme that links glycolysis to the tricarboxylic acid (TCA) cycle by converting pyruvate to Acetyl-CoA [25]. Our data shows that PDH activity tends to be elevated in the ventricles compared to the atria (Fig. 2E), possibly suggesting higher glycolysis in the ventricles compared to the atria.

Chamber-specific profiles were observed in energy production-related acylcarnitine abundances. Acylcarnitines, which play a key role in fatty acid β-oxidation and, thus, in cardiac energy production, displayed distinct patterns between the atria and ventricles. Clear clustering of acylcarnitines was evident, with the most pronounced differences related to acyl chain length (Fig. 2F). Acylcarnitines with fewer than ten carbons in their acyl chains were more abundant in ventricles, suggesting a cutoff at a chain length of ten carbons, beyond which acylcarnitine's abundance does not follow the same trend. For example, both acylcarnitine 5:0 isomers exhibited significantly higher levels in both the left and right ventricles compared to the corresponding atria (Fig. 2F). Additionally, the carnitine backbone, without an attached fatty acid, clustered with long-chain acylcarnitines and was significantly more abundant in the left atrium than the left ventricle. Lastly, despite the unique abundance patterns of acylcarnitine 2:0 and carnitine, the overall metabolic profile of acylcarnitines illustrated differences between ventricle and atria.

**Supplementary Fig. 1:**
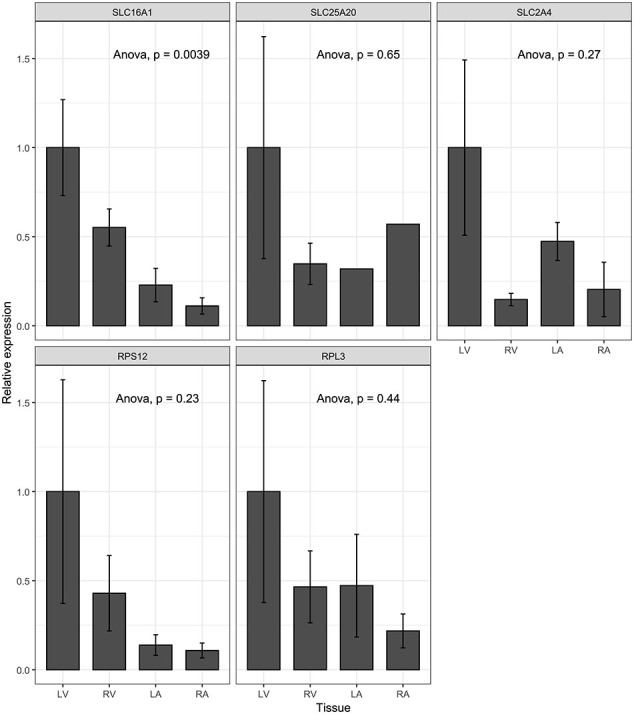
Gene expression seems to be higher in the left ventricle in qPCR results for selected genes. A. Solute Carrier Family 16 Member 1 (SLC16A1) B. Solute Carrier Family 25 Member 20 (SLC25A20) C. Solute Carrier Family 2 Member 4 (SLC2A4) D. Ribosomal Protein S12 (RPS12) E. Ribosomal Protein L3 (RPL3). Legend: (*, p < 0.05).

As these results indicate a large difference in energy metabolism between the chambers, we analysed gene expression for selected genes playing a vital role in energy substrate transportation in the heart. We selected Solute Carrier Family 16 Member 1 (SLC16A1), Solute Carrier Family 25 Member 20 (SLC25A20), and Solute Carrier Family 2 Member 4 (SLC2A4) to be analysed. These genes produce proteins Monocarboxylate Transporter 1, Mitochondrial Carnitine/Acylcarnitine Carrier Protein, and Insulin-Responsive Glucose Transporter Type 4, respectively. A significantly higher expression of SLC16A1 was detected in the ventricles compared to the atria (Supplementary material online, Fig. S1A). No difference was observed in the expression for genes SLC25A20 and SLC2A4 (Supplementary material online, Fig. S1B–C). Still, together with metabolomics results, higher expression of the gene related to energy substrate transportation, SLC16A1, suggests higher energy substrate capacity in the left ventricle.

### 3.3 Redox states vary across heart chambers

**Fig. 3:**
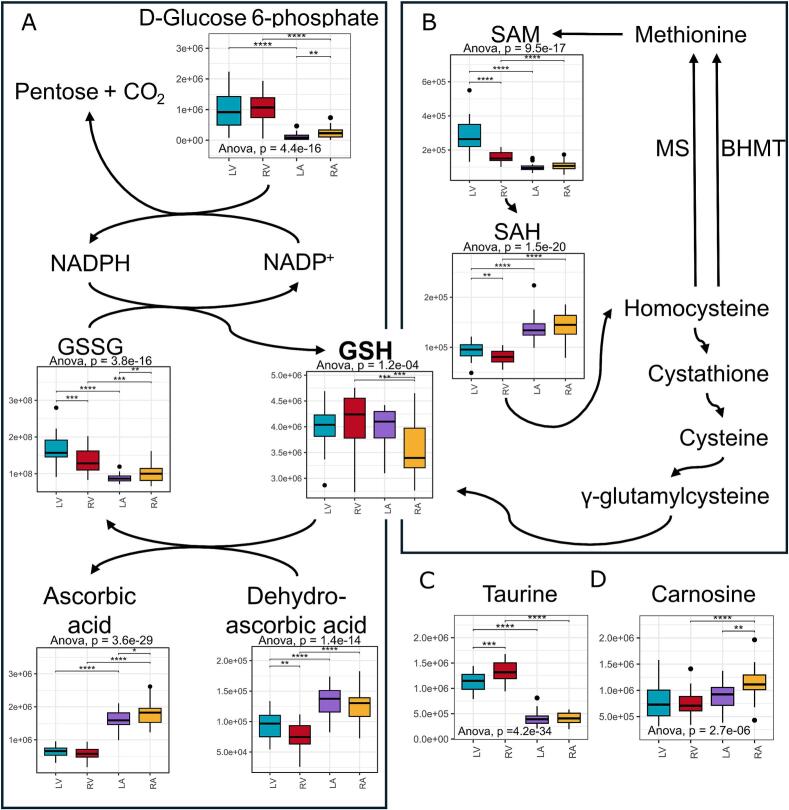
Heart chambers differ in several redox balance-related metabolites and pathways (n = 30 per group). (*A*) Ascorbic acid and glutathione in the cellular antioxidant cascade. Glutathione reductases catalyse the conversion of oxidised glutathione back to glutathione, which can further reduce dehydroascorbic acid back to ascorbic acid. (*B*) SAM and SAH are key substrates in glutathione synthesis on the transsulfuration pathway. (*C–D*) Redox balance-related metabolites had different abundances in chambers. Legend: (*, p < 0.05), (**, p < 0.01), (***, p < 0.001), and (****, p < 1e−04) in *t*-test, unadjusted. LV = left ventricle, RV = right ventricle, LA = left atrium, RA = right atrium, NADP^+^ = Nicotinamide adenine dinucleotide phosphate, NADPH = NADP + hydrogen, GSH = glutathione, GSSG = Oxidised GSH, SAM = S-adenosylmethionine, SAH = S-adenosylhomocysteine, BHMT = betaine-homocysteine methyltransferase, MS = methionine synthase.

**Supplementary Fig. 2:**
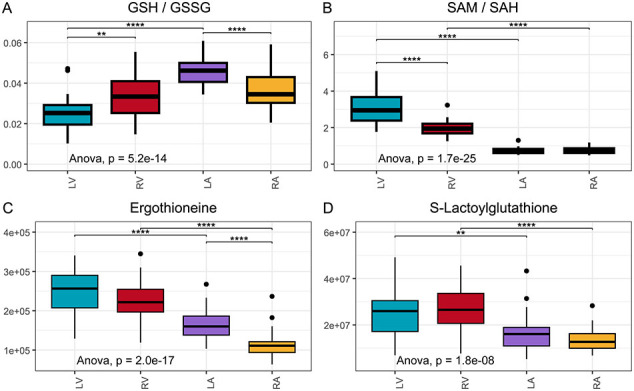
Antioxidation-related metabolites had a higher abundance in ventricles. Legend: Legend: (*, p < 0.05), (**, p < 0.01), (***, p < 0.001), and (****, p < 1e−04).

Consistent with the observed differences in metabolites linked to mitochondrial respiration, we found distinct variations in metabolite abundance between heart chambers, including those involved in redox reactions. Notably, glutathione, a critical antioxidant, was significantly more abundant in the right ventricle than in the right atrium (Fig. 3). The higher relative ratio of glutathione to its oxidised form (Supplementary material online, Fig. S2A) in the atria may reflect lower reactive oxygen species (ROS) concentrations, which could enhance the buffering capacity against oxidative stress, particularly in the atria. In contrast, the higher energy demand in the ventricles increases mitochondrial workload and susceptibility to electron leakage, thereby necessitating greater antioxidant utilisation in the ventricles.

In this study, ascorbic acid and its oxidised form, dehydroascorbic acid, were significantly more abundant in the atria compared to the ventricles (Fig. 3A). Additionally, results demonstrate that other oxidative stress-related metabolites, including S-adenosylmethionine (SAM) and its demethylated form, S-adenosylhomocysteine (SAH), both key substrates of the transsulfuration pathway, are differentially distributed between the atria and ventricles (Fig. 3B). Specifically, SAM abundances were significantly higher in the ventricles compared to the atria on both sides of the heart, and the lower SAH abundances in the ventricles resulted in a higher SAM to SAH ratio (Supplementary material online, Fig. S2B). This differential distribution of SAM and SAH suggests enhanced antioxidant capacity in the ventricles through the transsulfuration pathway, contributing to a greater antioxidant buffer and potentially offering increased protection against oxidative stress in the ventricles (Fig. 3B).

Other metabolites associated with antioxidant activity indicate an elevated antioxidant demand in the ventricles. For instance, taurine and FAD were more abundant in the ventricles (Figs. 1C, D, 3C, 2D), while carnosine—a dipeptide composed of beta-alanine and histidine with notable antioxidant properties—was more concentrated in the atria (Fig. 3D). Additionally, ergothioneine and S-lactoylglutathione, a precursor to glutathione, exhibited higher levels in the ventricles (Supplementary material online, Fig. S2C–D), further supporting the higher antioxidant requirement in the ventricles.

### 3.4 Heart chambers have differences in protein synthesis and degradation dynamics

**Fig. 4:**
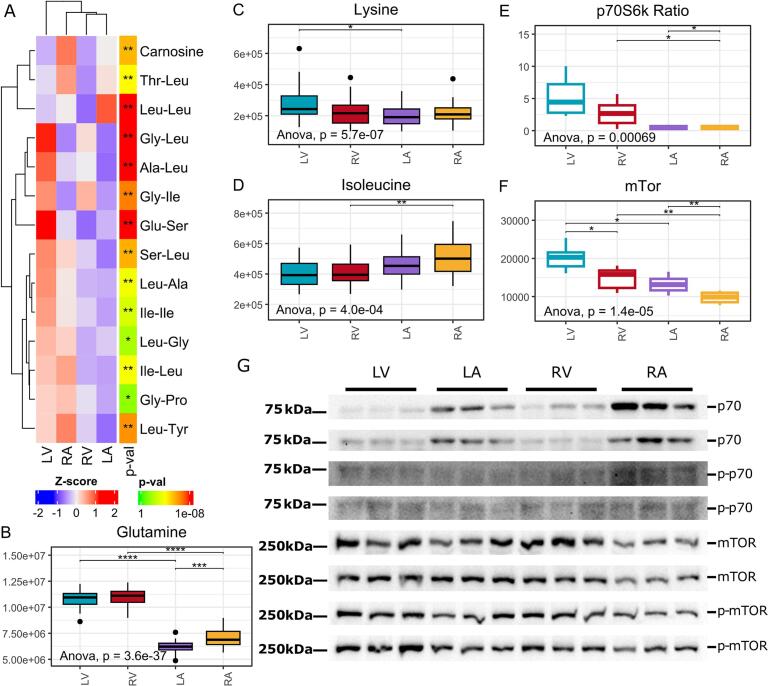
Protein turnover-related metabolites, such as amino acids and dipeptides are differently abundant in the heart chambers. (*A*) The heatmap shows the distribution of dipeptides in the heart chambers (n = 30 per group). (*, p < 0.05), (**, q < 0.05) in ANOVA, unadjusted and adjusted. (*B–D*) An abundance of amino acids varies between chambers (n = 30 per group). (*E*) The ratio between phosphorylated p70S6k and p70S6k. (*F*) mTor intensity in the chambers. (*G*) Western blot analysis of ribosomal protein kinase S6, mammalian target of rapamycin, and their phosphorylated forms in chambers (*n* = 6 per group). Legend: (*B–F*) (*, p < 0.05), (**, p < 0.01), (***, p < 0.001), and (****, p < 1e−04) in *t*-test, unadjusted. LV = left ventricle, RV = right ventricle, LA = left atrium, RA = right atrium, mTor = mammalian target of rapamycin, p70S6K = ribosomal protein kinase S6.

Following the observed differences in energy metabolism and redox status, we hypothesised that protein degradation rates might be elevated in the ventricles compared to the atria. Since currently known enzymatic pathways can synthesise only a limited number of dipeptides, such as carnosine and anserine [26], it can be inferred that the presence of other dipeptides in tissues primarily results from protein degradation rather than endogenous synthesis. Thus supporting our degradation hypothesis, dipeptide abundance varied significantly across the heart chambers (Fig. 4A). Among the 14 identified dipeptides, dileucine uniquely displayed lower abundance in the left ventricle compared to the left atrium, while 10 out of 14 dipeptides were significantly more abundant in the left ventricle than in the left atrium (Fig. 4A). Additionally, the dipeptides glycylleucine and glycylisoleucine were significantly more abundant in the ventricles than in the atria, while carnosine was more abundant in the right atrium than in the right ventricle and left atrium, with no significant difference observed on the left side (Fig. 4A). Together, these results suggest increased protein degradation in the left ventricle, potentially driven by the greater mechanical strain and need for protein repair in this chamber.

In contrast, the free amino acid profiles exhibited distinct patterns between the heart chambers, with 13 out of 15 amino acids differing significantly between the left ventricle and left atrium. Notably, only glutamine and lysine were more abundant in the left ventricle (Figs. 1D, E, 4B–C), indicating a unique amino acid distribution pattern likely supporting increased protein synthesis and a higher intrinsic demand for amino acids in the left ventricle.

**Supplementary Fig. 3:**
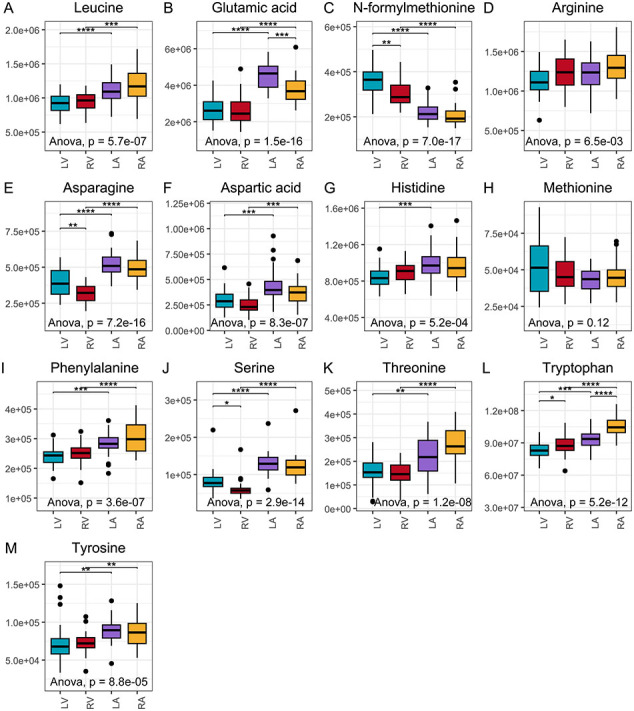
The abundance pattern of amino acids varies between the chambers. Legend: (*, p < 0.05), (**, p < 0.01), (***, p < 0.001), and (****, p < 1e−04).

Most free amino acids, including branched-chain amino acids (BCAAs) such as isoleucine and leucine, were significantly more abundant in the atria on both sides of the heart (Fig. 4D and Supplementary material online, Fig. S3A). The exception was glutamine, which was more concentrated in the ventricles (Fig. 4B), whereas glutamic acid showed the opposite pattern, with higher levels in the atria (Supplementary material online, Fig. S3B). Additionally, other metabolites with structural properties similar to human proteogenic amino acids, such as taurine and *N*-formylmethionine, of which later initiate protein synthesis in mitochondrial translation [27], were significantly more abundant in the ventricles than in the atria (Fig. 3C, Supplementary material online, Fig. S3C). Collectively, these findings indicate chamber-specific differences in amino acid distribution (Supplementary material online, Fig. S3), suggesting specialised metabolic requirements for protein synthesis across heart compartments and/or amino acid degradation in energy production.

Nucleoside monophosphates, integrated into energy metabolism and protein synthesis, exhibited distinct patterns of chamber-specific abundance. Notably, purine nucleotides such as AMP and ADP were significantly enriched in the left ventricle and right atrium, with comparatively lower levels detected in the left atrium and right ventricle (Fig. 2D). Additionally, other nucleotides involved in protein synthesis pathways, such as inosine monophosphate (IMP) and the pyrimidine nucleotide uridine monophosphate (UMP), also showed distinct chamber-specific distributions. IMP, a precursor in purine nucleotide biosynthesis, was found at significantly higher abundance within the atria (Fig. 2D). Similarly, UMP, which participates in RNA synthesis and pyrimidine metabolism, demonstrated elevated levels in the left ventricle and right atrium, mirroring the distribution pattern observed for AMP and ADP (Fig. 2D). These results suggest regionally specialised roles for protein synthesis pathways within the heart chambers, potentially supporting differential transcriptional and translational demands in protein synthesis-related pathway activities across heart chambers.

To further explore the pattern of protein turnover, we performed a Western blot analysis for the mammalian target of rapamycin (mTOR) and ribosomal protein kinase S6 (p70S6K) to evaluate differences in the protein synthesis pathway in the heart. Here, both mTOR and p70S6K revealed chamber-specific differences. The ratio of phosphorylated p70S6K to total p70S6K was more than fivefold higher in the ventricles compared to the atria, with the left ventricle showing the highest ratio (5.3), followed by the right ventricle (2.7), and the atria (0.5 in both right atrium and left atrium), indicating higher protein synthesis in the ventricles (Fig. 4E, G). Although the phosphorylated mTOR to total mTOR ratio did not indicate higher enzymatic activity in the ventricles, the total abundance of the active form of mTOR was significantly higher in the ventricles (Fig. 4F, G), further supporting the observed differences in protein turnover. Additionally, we analysed the expression of ribosomal protein genes Ribosomal Protein S12 (RPS12) and Ribosomal Protein L3 (RPL3) using qPCR, based on the observed amino acid and dipeptide metabolomic differences. As expected, both genes exhibited higher expression in the ventricles (Supplementary material online, Fig. S1D, E), thus further supporting higher protein turnover in the left ventricle compared to other chambers.

### 3.5 The difference in metabolite abundance results in variation in pathway activity

**Fig. 5:**
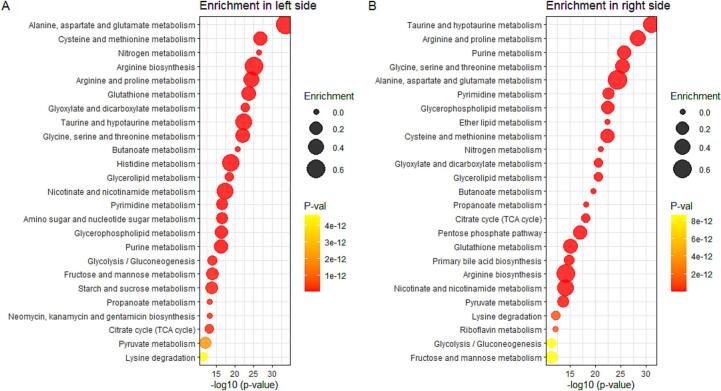
Energy and amino acid-related pathways are more active in ventricles. (A) Activated pathways in the left ventricle compared to the left atrium (n = 30 per group). (B) Activated pathways in the right ventricle compared to the right atrium (n = 30 per group).

Through metabolomics pathway analysis, significant disparities in metabolic pathways were identified, highlighting an extensive range of differentially abundant metabolites. Metabolite profiling, when mapped to the pig (*Sus Scrofa*) Kyoto Encyclopedia of Genes and Genomes (KEGG) database, revealed distinct metabolic pathway variations, with pronounced differences in taurine metabolism (Fig. 5A, B) and several energy-related metabolic pathways. Specifically, key pathways in energy metabolism such as the citrate cycle and glycolysis/gluconeogenesis exhibited differential activity between the left ventricle and atrium. A more in-depth examination of given pathways indicated that metabolites like glucose-6-phosphate, fructose-6-phosphate, and succinic acid are instrumental in influencing the observed variations in energy metabolism.

In contrast, amino acid abundances play a substantial role in modulating multiple pathways. For example, the glyoxylate and dicarboxylate metabolism pathways showed enrichment driven by variances in serine, glutamic acid, and glutamine levels. Furthermore, pathways include 1. arginine and proline metabolism, 2. glycine, serine, and threonine metabolism, and 3. alanine, aspartate, and glutamate metabolism, ranked among the six most differentially activated pathways on the right side of the heart (Fig. 5B). Lastly, glutathione metabolism emerged as a distinctively active pathway across both the left and right cardiac regions (Fig. 5A, B), underscoring an increased demand for redox homeostasis, particularly within the left ventricle.

## 4 Discussion

In this study, we conducted a comprehensive metabolomics analysis using LC-MS, complemented by focused gene expression and protein analyses, across healthy heart compartments, left and right ventricles, and atria, using the large mammalian animal model, the pig. Previous cardiac metabolomics studies have largely focused on pathological conditions [28], relied solely on blood samples [[29], [30], [31], [32]], or been limited to small animal models such as mice [17]. However, it is widely known that rodent models replicate human physiology poorly, and therefore, it is imperative to deepen the studies with better models, such as pig [18]. To the best of our knowledge, this is the first study to present a comprehensive overview of chamber-specific metabolite profiles in healthy pig hearts.

Given the higher energy demands of the ventricles, we anticipated an enrichment in energy metabolism pathways. As expected, metabolites related to energy production, including electron transporters such as FAD and NAD^+^, were more abundant in the ventricles. This aligns with the ventricles' greater mitochondrial respiratory capacity, e.g. fatty acid oxidation [33,34]. Acylcarnitines, essential for fatty acid oxidation, were differentially abundant between chambers: ventricles had higher short-chain acylcarnitines, while long-chain acylcarnitines were equally abundant or slightly more abundant in atria. The predominance of shorter acylcarnitines in ventricles may be attributed to higher energy reserves and/or the faster mitochondrial transport of short-chain acylcarnitines. This distinct distribution of acylcarnitines with varying chain lengths likely reflects the ventricles' greater capacity for mitochondrial fatty acid oxidation, either alone or in combination with peroxisomal metabolism. Ventricular cardiomyocytes contain more peroxisomes, which primarily process longer acylcarnitines [35].

Interestingly, other measurements related to energy metabolism showed a trend towards higher activity in the ventricles. For example, pyruvate dehydrogenase, a key enzyme connecting glycolysis to the citric acid cycle in mitochondria, tended to have a higher level in ventricles, and gene expression analysis indicated that genes related to energy substrate transportation were more expressed in the left ventricle. Previously, acylcarnitine 16:0 has been associated with lower PDH activity and pyruvate levels in the heart [14,36]. Our results demonstrated slightly less PDH activity in atria, even though acylcarnitine 16:0 had no difference between the chambers. However, the difference in pyruvate dehydrogenase between ventricles and atria was not as pronounced as expected. Still, hexose-phosphates were consistently more abundant in ventricles than atria, suggesting higher glycolysis, thus leaving open questions whether glucose is used anaerobically in cardiac energy production or does it propose higher energy reserve together with higher acylcarnitine levels.

Interestingly, AMP and ADP were more abundant in the right atrium and left ventricle, further suggesting a differential energy status between the chambers. Our instrumentation favoured broad-spectrum metabolite detection but did not identify ATP, and is suboptimal for AMP and ADP, preventing a comprehensive evaluation of energy metabolism. Despite the general understanding that fatty acids serve as the primary energy source [15], the metabolic versatility observed in the left ventricle underscores the importance of utilising diverse energy sources under varying physiological conditions [37].

Our results indicate distinct redox states across the heart chambers. Ascorbic acid (Vitamin C), a key antioxidant [38], was more abundant in the atria, while its oxidised form, dehydroascorbic acid, was also more abundant in the atria, particularly in the right atrium. Although pigs can synthesise ascorbic acid [39], it mainly occurs during the gestation period [40]. Furthermore, ascorbic acid is primarily synthesised in the liver [40], making it similarly available to all heart chambers. Hence, varying levels of ascorbic acid abundance likely suggest a difference in need and consumption of ventricle antioxidants. Less likely, a higher antioxidant requirement or enhanced ROS protection in the atria could be suggested. Interestingly, the abundance level of glutathione was the highest in the right ventricle and lowest in the right atrium, possibly suggesting that dehydroascorbic acid could be reduced spontaneously or enzymatically with glutathione [41]. Furthermore, the left ventricle exhibited the lowest glutathione to oxidised glutathione ratio, indicating a higher consumption of antioxidant agents in the left ventricle and possibly reduced capacity to respond to externally caused elevation of oxidative stress compared to other chambers. These results align with varying levels of antioxidants in the ventricles, possibly supporting greater oxidative consumption, especially in the left ventricle.

Taurine, known for its antioxidant properties [42] and potential to act also as an anti-inflammatory agent [43], was more abundant in the ventricles. A greater abundance of taurine in the ventricles might also indicate an adaptive mechanism to cope with the elevated energy requirements and oxidative stress inherent to ventricular activity [44]. Taurine's role in calcium homeostasis [45] and its role in other key mechanisms in the heart, underscore the ventricles' reliance on diverse substrates to meet physiological demands.

Ergothioneine was demonstrated to be higher in the ventricles in our analysis. Given that the metabolite has antioxidant activities, it likely contributes to higher antioxidant activity in the ventricles. Moreover, it was decreased previously in the heart failure model [17], emphasising the difference between heart models. In contrast, carnosine, which enhances glutathione levels and exhibits antioxidant properties [46,47], was more abundant in the right atrium, supporting the theory of higher consumption of antioxidants in the ventricles. Additionally, carnosine has been associated with reduced cell renewal by mTOR inhibition and protection against myocardial ischemia-reperfusion injury [48,49]. The lower antioxidant ratios, e.g. between glutathione and oxidised glutathione in the left ventricle, together with known higher protein turnover and DNA content [50], suggest that a higher ROS load may drive a higher need for protein synthesis in the left ventricle. The enrichment of purine and pyrimidine metabolism pathways in the ventricles further supports the perception that cell synthesis and protein turnover are more active in the ventricles compared to the atria.

Our study found significant differences in amino acid metabolism between heart chambers, suggesting more active protein synthesis in the ventricles, likely due to greater physiological demand. The ventricles exhibited higher levels of protein synthesis-related proteins such as p70 and mTOR, which are critical for cell growth and protein synthesis [51,52]. Additionally, ribosomal gene expression, especially RPL3 and RPS12, which are integral components of ribosomes [53,54], was higher in the ventricles, indicating increased protein synthesis. While previous studies have utilised heart failure models in rats to investigate protein turnover rates in the heart [55], to our knowledge, protein turnover has not been specifically examined in different cardiac regions.

Unexpectedly, BCAAs leucine and isoleucine were more abundant in the atria, despite their known role in mTOR activation and protein synthesis [56]. This paradox suggests that BCAAs in the atria may serve different regulatory functions or contribute to specific metabolic processes beyond protein synthesis. In contrast, other amino acids, such as glutamine, had a higher abundance in the ventricles. Glutamine is known for its contribution to energy metabolism [57]. Thus, the higher abundance of glutamine in the ventricles may reflect an adaptive response to the increased energy demands and oxidative stress associated with ventricular function [44]. Nevertheless, the interplay between taurine, glutamine, and BCAAs suggests a complex regulatory network that supports protein synthesis and energy metabolism, particularly in the left ventricle, where metabolic demands are heightened. Moreover, *N*-formylmethionine had a higher abundance in the ventricles. It is known as a mitochondrial translation marker [58], indicating higher synthesis of mitochondrial protein and/or turnover of mitochondria in the ventricles.

Moreover, dileucine has been associated with higher protein turnover stimulation than leucine in muscle [59]. Although we did not isolate cardiomyocytes from non-cardiomyocytes, the importance of dipeptides in myocardial energetics has been demonstrated by utilising the α-MHC promoter, which is highly specific for cardiomyocytes [60]. Since only a few dipeptides are endogenously synthesised [26], rest of the dipeptides primarily result from protein degradation. Thus, our findings suggest chamber-specific variations in dipeptide abundance across different heart compartments. This metabolite pattern may reflect the elevated mechanical workload and structural stress in the ventricles [61], potentially resulting in higher protein degradation and dipeptide levels in the left ventricle. Although our methodology allows us to evaluate only relative abundances, which don't necessarily describe the difference in pathway or process, the variations in dipeptide levels could stem from the higher workload in the ventricles, leading to increased oxidative stress, a greater abundance of antioxidants, and consequently higher protein degradation. This degradation necessitates elevated protein synthesis and turnover.

Understanding chamber-specific metabolic profiles could provide valuable insights into cardiac disease mechanisms. As our study was focused on healthy hearts, we couldn't assess the injury or disease response, which has been demonstrated to alter the metabolite profiles significantly [62,63]. Nevertheless, integrating this knowledge with biomarker studies [64] could pave the way for more targeted therapeutic and diagnostic strategies, potentially improving patient outcomes. Future research into the roles of the chamber-specific metabolome in cardiac function is needed to elucidate how metabolites contribute to altered production, degradation, uptake, or the time-dependent fluctuations in given processes, ideally using multi-time point analyses.

In conclusion, this study provides the first comprehensive chamber-specific metabolomic profile of healthy pig hearts. The results reveal significant differences in energy metabolites, such as higher hexose-phosphates, and the opposite levels of redox metabolites, for example, higher ascorbic acid abundance in atria. These findings offer new insights into the metabolic demands of different cardiac regions and highlight the potential for using metabolomics to model early cardiac dysfunction. Further research is needed to explore how these chamber-specific differences manifest under chamber-specific pathological conditions and to determine whether these chamber-specific differences persist in diseased states and in humans.

The following are the supplementary data related to this article.

Supplementary Table 1 Metabolite profiling results from the heart tissue. Supplementary Table 1

## CRediT authorship contribution statement

**Retu Haikonen:** Writing – original draft, Visualization, Formal analysis. **Topi Meuronen:** Writing – review & editing, Data curation, Conceptualization. **Ville Koistinen:** Writing – review & editing, Visualization, Supervision. **Olli Kärkkäinen:** Writing – review & editing, Supervision. **Tomi Tuomainen:** Writing – review & editing, Formal analysis. **Gloria I Solano-Aguilar:** Writing – review & editing, Data curation. **Joseph F. Urban:** Writing – review & editing, Supervision. **Marko Lehtonen:** Writing – review & editing, Data curation. **Pasi Tavi:** Supervision. **Kati Hanhineva:** Writing – original draft, Supervision, Project administration.

## Declaration of Generative AI and AI-assisted technologies in the writing process

During the preparation of this work, the author(s) used ChatGPT (GPT-4, OpenAI) in order to improve the clarity in the discussion. After using this tool/service, the author(s) reviewed and edited the content as needed and take(s) full responsibility for the content of the publication.

## Funding

This work was supported by the 10.13039/100007753University of Eastern Finland; grants from the Research Council of Finland [#321716 to K.H., #365298 and #325510 to P.T.]; 10.13039/501100004022Jenny and Antti Wihuri Foundation [R.H.]; the Lantmännen Foundation [2020H025 to R.H. and K.H., 2022H045 to V.M.K and K.H.]; 10.13039/501100004012Jane and Aatos Erkko Foundation [K.H.]; and Sigrid Juselius Foundation [P.T.].

## Declaration of competing interest

T.M., V.M.K., O.K., and K.H. are affiliated with Afekta Technologies Ltd.

## Data Availability

The data supporting the findings presented in this study are available within the article and its Supplementary material.
